# Analysis of the effect of nursing care based on action research method on the prevention of postoperative lymphedema in breast cancer patients

**DOI:** 10.1097/MD.0000000000036743

**Published:** 2023-12-29

**Authors:** Weijuan Yang, Li Yang, Shuangwei Mao, Dandan Liu, Lili Wang

**Affiliations:** a Breast Surgery Department, Jiangsu Province Hospital, and the First Affiliated Hospital of Nanjing Medical University, Nanjing, China.

**Keywords:** action research approach, anxiety, depression, life skills, lymphoedema, post-breast cancer

## Abstract

**Rationale:**

In recent times, the pervasive adoption of the action research method has garnered substantial attention both domestically and internationally. Its integration has traversed various domains of nursing research, nursing education, and nursing practice, yielding commendable outcomes. However, a notable gap persists, as this method remains untapped in the realm of nursing care concerning the prevention of postoperative lymphedema in breast cancer patients.

**Diagnosis:**

To employ the action research methodology in the context of patients undergoing axillary lymph node dissection surgery for breast cancer, aiming to investigate its impact on mitigating postoperative lymphedema and assessing its influence on the patient’s quality of life, as well as levels of anxiety and depression postoperatively.

**Intervention:**

The study focused on breast cancer patients admitted to our hospital from January 2022 to December 2022. Among them, 44 patients from January to June constituted the control group, while 44 patients from July to December comprised the observation group. Conventional nursing measures were applied to the control group, whereas the observation group received nursing interventions rooted in the action research method. A comparative analysis was conducted between the 2 groups, assessing the incidence of postoperative lymphedema, daily life ability, as well as levels of anxiety and depression.

**Outcomes:**

The prevalence of edema was notably reduced in the observation group (20.93%) compared to the control group (42.22%), with a statistically significant difference. Throughout the study, patients in both groups exhibited increased Barthel Index Scale scores from the study’s initiation, and the scores for the observation group surpassed those of the control group, reaching statistical significance (*P* < .05). Furthermore, by the study’s conclusion, anxiety and depression scores for patients in both groups were diminished compared to the study’s commencement, and the observation group demonstrated significantly lower scores in anxiety and depression compared to the control group (*P* < .05).

**Lessons:**

The implementation of nursing care grounded in the action research methodology exhibits a capacity to diminish both the occurrence and intensity of postoperative lymphedema in breast cancer patients. Concurrently, it enhances the patients’ daily life functionality and mitigates symptoms of anxiety and depression.

## 1. Introduction

Breast cancer stands as the foremost prevalent malignancy profoundly impacting women’s health. Reports indicate that newly diagnosed cases of breast cancer in women across Europe and the United States constitute approximately one-third of all cancer incidences.^[1]^ According to statistics, the number of new cases of female breast cancer in China in 2021 is about 420,000, accounting for 9.1% of all cancers.^[2]^ Breast cancer treatment is currently based on surgery, but surgery and subsequent treatment can cause damage to the axillary lymphatic vessels. Breast cancer-related lymphedema (BCRL) is a severe postoperative complication of breast cancer. The incidence of BCRL varies widely (14–60%) as the diagnostic criteria for lymphedema vary, and the length of follow-up varies in previous studies.^[3]^ It primarily arises from the perturbation of the typical physiological anatomy of the lymphatic system, encompassing treatments that impede the customary drainage of protein-rich lymphatic fluid. This obstruction leads to the partial accumulation of fluid within tissues, resulting in swelling observed in the arms, shoulders, hands, breasts, and related areas of the torso.^[4]^ Studies have shown that the swelling, pain, and numbness in the limbs caused by BCRL can severely interfere with the patient’s sleep, activities, sexual behavior, and other daily activities, reducing their quality of life.^[5]^ BCRL requires long-term or lifelong treatment and places a significant financial burden on the patient, family, and society, including the direct financial burden of comprehensive swelling treatment, elastic cuffs, and pneumatic compression therapy and the indirect financial burden of low productivity due to the development of edema.^[6]^ Studies have shown that lymphoedema tends to develop into a recurrent, persistent pathology if not recognized and intervened in early.^[7]^ It also contributes significantly to the incidence of cellulitis due to the fluid-rich tissue spaces and poor lymphatic drainage in swollen limbs.^[8]^ Cellulitis is characterized by red and warm skin, localized pain, poorly defined borders, rapid deterioration, and accelerated reactive fibrosis of the skin, with the onset of inflammation further accelerating the progression of edema, creating a vicious circle.^[9]^ Severe infections may also lead to the development of bacteremia and may even result in serious consequences in the form of amputation.^[10]^

In light of the evolving array of treatment modalities, including surgical interventions and adjuvant radiotherapy, the annual augmentation in the survival rate of breast cancer is evident. The attention of both breast cancer survivors and public health entities is progressively pivoting from mere survival metrics to the nuanced exploration of quality of life. The emergence of BRCL stands out as a substantial factor contributing to the diminishing quality of life among breast cancer survivors. Preventive measures, therapeutic interventions, and ongoing care strategies for lymphedema have achieved a relatively advanced status internationally. Corresponding intervention studies have predominantly concentrated on information support and the formulation of self-management plans. Ligabue MB et al devised a one-month training program aimed at instructing female lymphedema patients in self-administered comprehensive decongestive therapy. The outcomes demonstrated a reduction in pain and swelling in the affected limb, coupled with a noteworthy improvement in self-care.^[11]^ Ostby et al delivered health education pertaining to lymphedema treatment and risk mitigation, tailoring the content to the specific requirements of breast cancer survivors with lymphedema as identified by healthcare providers.^[9]^ This approach addressed the physical, psychological, and social barriers to self-management. The provision of educational support to lymphedema patients resulted in enhanced self-care capabilities. In the Chinese context, the predominant focus of studies has centered on evaluating and treating lymphedema, with limited attention devoted to researching its prevention and management. Although recent years have witnessed an emergence of studies exploring lymphedema prevention, most tend to concentrate on the efficacy of individual measures. However, a comprehensive evaluation of preventive behaviors is notably absent, and the majority of interventions occur within hospital settings. Consequently, assessing patients’ sustained commitment to lymphedema prevention and their adherence to preventive measures remains imperative. Notably, investigations reveal a considerable deficit in breast cancer patients’ knowledge regarding lymphedema prevention, underscoring the adverse impact of insufficient professional supervision on postoperative lymphedema prevention.^[12]^ In a survey encompassing 135 patients undergoing mastectomy for breast cancer, Davies et al observed that a substantial 89.6% exhibited a lack of awareness concerning the risk factors associated with the onset of BCRL, coupled with insufficient knowledge regarding the management of BCRL.^[13]^ A parallel investigation by Jeffs et al delineated a prevailing dearth of awareness among clinical healthcare professionals regarding the gravity of BCRL. This knowledge gap manifested in inadequate communication with BCRL patients, impeding their capacity to promptly discern and address early symptoms. Moreover, healthcare professionals displayed deficiencies in knowledge and skills related to edema care, thereby contributing to an overall suboptimal outcome in the control of edema.^[14]^ Consequently, the identification of efficacious strategies to prevent lymphedema has assumed paramount significance as an integral facet of postoperative self-management for individuals diagnosed with breast cancer.

Action research represents a contemporary educational research methodology that has gained considerable traction in Western nations since the 1970s. Its integration into the nursing domain occurred in the 1990s, marking widespread adoption. The inception of the term “action research” can be traced back to the period from 1933 to 1945, when Coller and colleagues initially introduced the concept. Subsequently, in 1944, Lewin not only coined the term “action research” but also formulated the foundational framework for this methodology. Lewin seminal contributions encompass the articulation of the spiral cycle model of action research, encompassing 4 fundamental phases: planning, action, observation, and reflection. This model serves as a theoretical underpinning for the operationalization of action research in diverse contexts, including nursing research.^[15]^ Within the nursing domain, Webb C characterizes action research as a nuanced research methodology involving small-scale interventions in authentic professional activities. This method entails a meticulous examination of the outcomes stemming from such interventions, thereby tightly integrating the resolution of practical workplace challenges with the research process.^[16]^ Notably, in the realm of action research, practitioners transition from being mere objects of investigation to active subjects. They engage in a dynamic and iterative process, mobilizing their initiative to address workplace issues through a perpetual cycle of planning, action, observation, and reflection. This cyclical sequence underscores the iterative nature of action research, wherein each phase informs and shapes the subsequent stages, fostering a continuous refinement of solutions to practical challenges encountered in the professional milieu. In contemporary contexts, the widespread adoption of the action research methodology is discernible on both domestic and international fronts. This approach has infiltrated various realms of nursing, spanning research, education, and practice, demonstrating its effectiveness through commendable outcomes. Noguchhi et al used an action research approach in the intensive care unit, which enabled nurses to consider their actions from the patient’s perspective, to notice better the communication intentions of lightly sedated mechanically ventilated patients, and to improve patient satisfaction, prompting a change from intensive care unit-based nursing practice to patient-centered care.^[17]^ Theobald et al used an action research approach to reveal the interactions, challenges, and outcomes when implementing an inquiry-based learning approach to support the development of student’s clinical reasoning and the ability to think like a nurse.^[18]^ Maindal et al used an action research approach to manage the lifestyle of prediabetic patients and designed an individualized lifestyle intervention for early diabetes for diabetic patients.^[19]^ Limited research has been documented regarding the utilization of the action research method for the prevention of postoperative lymphedema in breast cancer patients. This study posits the application of the action research method to enhance the capacity of breast cancer patients to avert lymphedema, thereby mitigating the risk of its onset. The overarching objective is to ameliorate patients’ self-care capabilities and alleviate negative emotional states, including anxiety and depression.

## 2. Materials and methods

### 2.1. General information

The study cohort comprised individuals diagnosed with breast cancer who were admitted to our institution from January 2022 to December 2022. Inclusion criteria encompassed individuals aged 18 years and above, confirmed breast cancer diagnosis through imaging or pathology, all having undergone axillary lymph node dissection. Additionally, participants were required to be conscious, possess the ability to communicate, read, understand, and demonstrate awareness of the study, volunteering for participation. Exclusion criteria: upper limb disability or venous thrombosis; cardiogenic, nephrogenic, or other causes of edema; combined organ dysfunction or other tumors. The sample size was calculated according to the formula n1 = n1 = n2 = 2×{(Z_ɑ/2_ + Z_β_)×σ/δ}², based on the comparison of the means of the 2 samples. Applying a significance level (α) of 0.05 and a power level (β) of 0.1 in a two-sided test, critical values were verified by consulting the table, resulting in Z_ɑ/2_ = 1.96 and Z_β_ = 1.282. With known values of σ = 4.02 and δ = 3.26, substitution into the formula yielded n1 = n2 = 32. Accordingly, the preliminary determination suggested a sample size of 64 cases. However, accounting for a potential 20% missed visit rate, a judicious adjustment led to the final sample size of 88 cases. This distribution comprised 44 patients observed from January to June, constituting the control group, and an equivalent number of patients observed from July to December, forming the observation group. The control group exhibited an average age of (53.16 ± 4.35) years, with educational distribution as follows: 18 cases (40.9%) with education up to junior high school, 20 cases (44.5%) with a high school education, and 6 cases (13.6%) with a college education or above. Regarding marital status, 40 cases (90.9%) were married, while 4 cases (9.1%) were either unmarried or divorced. TNM staging revealed 16 cases (36.4%) at stage 1, 21 cases (47.7%) at stage 2, and 7 cases (15.9%) at stage 3. Shoulder mobility on the affected side was measured at (170.93 ± 7.99)°. The observation group had an average age of (54.12 ± 4.88) years, with educational distribution as follows: 18 cases (40.9%) with junior high school education or below, 19 cases (43.2%) with high school education, and 7 cases (15.9%) with college education or above. In terms of marital status, 39 cases (88.6%) were married, while 5 cases (11.4%) were unmarried or divorced. The TNM stage distribution included 17 cases (38.6%) in stage 1, 19 cases (43.2%) in stage 2, and 8 cases (18.2%) in stage 3. Shoulder mobility on the affected side was measured at (170.23 ± 6.26)°. There were no statistically significant differences between the 2 groups at baseline in terms of age (*t* = 0.976, *P* = .332), education (*X*^2^ = 0.103, *P* = .950), marital status (*X*^2^ = 0.124, *P* = .725), TNM stage (*X*^2^ = 0.197, *P* = .906), and shoulder mobility (*t* = −0.456, *P* = .649).^[20,21]^ The observed differences were not statistically significant (*P* > .05). The study received approval from the hospital ethics committee, and all patients or their family members provided signed informed consent forms.

### 2.2. Methods

In the control group, conventional postoperative care protocols for breast cancer were applied. Patients received preoperative education encompassing information on the risk factors, clinical manifestations, and surgical precautions associated with breast cancer. Following surgery, patients were guided in the proper execution of functional exercises. Standard instructions were provided upon discharge, among other routine procedures. The observation group, building upon the control group’s framework, employed an action research methodology, the implementation of which is delineated as follows.

#### 2.2.1. The study team was established.

The team leader was a nursing staff member with 18 years of working experience and the title of chief nurse, while the deputy leader held 12 years of working experience and the title of deputy chief nurse. Additionally, the team included 5 nurse practitioners in charge and 3 deputy nurse practitioners, totaling 10 members. Before the intervention, all group members underwent a one-week theoretical lecture to familiarize themselves with the action research method and lymphedema following breast cancer surgery. At the conclusion of the lecture, all members underwent an assessment, and each member successfully passed. The study received ethical approval with the reference number: Jiangsu Provincial People’s Hospital 2023-SR-085.

#### 2.2.2. Methods implementation of a planned action-based approach to care.

The nursing intervention, rooted in the action research methodology, was administered over 2 distinct phases, each spanning 3 months, thereby encompassing a comprehensive 6-month intervention period. The intervention protocol consisted of sequential stages, encompassing planning, action, observation, and reflection. Subsequently, these stages were revisited through a process of replanning, reaction, reobservation, and reflection, thereby establishing a cohesive and iterative spiral cycle pattern.

##### 2.2.2.1. Plan.

① The team members understood the extent of patients’ knowledge related to postoperative lymphoedema after breast cancer through interviews, and understood the difficulties in preventing postoperative lymphoedema through communication with doctors, pointed out the main risk factors related to postoperative lymphoedema after breast cancer, and made a health education booklet on risk factors, preventive measures, lymphatic return exercises, and self-management methods to make patients aware of the importance of preventive measures and self-management in preventing lymphoedema. ② Establish a nurse-patient communication log to comprehend psychological challenges and uncertainties among patients, facilitating targeted guidance within the 24 hours preceding discharge.

##### 2.2.2.2. Actions.

① Distribute the postoperative lymphoedema prevention health education booklet after the patient returns to the ward, explaining the relevant postoperative precautions in a language that the patient can understand, and the patient understands the knowledge related to the prevention of postoperative lymphoedema. ② A weekly instructional session about postoperative lymphoedema is convened each Thursday afternoon. This forum serves a dual purpose: firstly, it serves to reinforce the content encapsulated within the postoperative lymphoedema prevention health education manual; secondly, it enhances the patients’ cognizance of postoperative self-management strategies. ③ Postoperative individuals receive notification regarding the establishment of a nurse–patient communication repository at the nurse station. This platform facilitates the expression of postoperative perplexities, apprehensions, and anxieties by patients through written entries in the designated nurse-patient communication book. The designated nurse-in-charge systematically reviews the nurse-patient register on a scheduled weekly basis, offering personalized guidance to patients who have recorded messages. This interaction aims to proffer tailored solutions, mitigate confusion, and alleviate anxiety and depression among the concerned individuals. ④ Scheduled lectures and patient assemblies are conducted bi-monthly. These organized sessions specifically tackle inquiries and issues extracted and synthesized from patients. The primary emphasis is on elucidating and resolving patients’ questions and uncertainties, aligning with a patient-centric approach.

##### 2.2.2.3. Observations and reflections.

Approximately 50% of the patient cohort exhibited a deficient grasp of the contents of the postoperative lymphoedema prevention health education booklet, coupled with limited comprehension of the significance of self-management, along with a notable deficit in patience and confidence. Concurrently, nearly 60% of patients manifested negative emotional states, such as anxiety and depression, stemming from concerns regarding their future capacity for self-care. A substantial 70% of patients encountered challenges in cooperating with the documentation process in the bedside doctor-patient communication book, displaying a notable resistance to its utilization. In response to the identified issues during the initial phase, the collaborative efforts of the research team and patients led to prompt discussions and reflections on the necessity for program adjustments. This proactive approach sought to address and modify the observed challenges from the inaugural stage, paving the way for their effective implementation in subsequent iterations of the program.

##### 2.2.2.4. Replanning.

Revising and adjusting the plan in the second round in light of the problems that arose in the first round. ① The health education booklet on postoperative lymphedema prevention is presented through a combination of illustrations and text, while the lymphatic reflux exercise is demonstrated via video. ② Utilizing the network platform, a WeChat public account has been established, accompanied by the creation of a WeChat exchange group. This facilitates the timely dissemination of knowledge and videos related to postoperative lymphedema prevention. ③ Implement the creation of a bedside nurse-patient communication book, designed to be promptly collected and securely stored post-discharge to safeguard patient privacy.④ Establish a psychological consultation group dedicated to monitoring and addressing the emotional well-being of patients. This initiative aims to promptly identify and address any negative emotions, ensuring the provision of effective psychological care. ⑤ Institute a comprehensive follow-up register, conducting weekly sessions lasting over 30 minutes each. During these sessions, guide patients in actively engaging in self-management, collaboratively formulate personalized self-management plans, emphasize adherence to long-term standardized treatment, and encourage regular follow-up to ensure ongoing care.

##### 2.2.2.5. Reaction.

① After surgery, patients received a visual rendition of the postoperative lymphedema prevention health education booklet. A demonstrative video illustrating lymphatic reflux exercises was presented, accompanied by bedside explanations and discussions. The comprehension level of patients was assessed through interactive question-and-answer sessions. ② Introduce the bedside nurse-patient communication book to patients, guiding them to record their thoughts promptly. The research team members engage in daily consultations, reviewing the nurse–patient communication book and providing personalized, one-to-one guidance. This process aims to enhance patients’ awareness of self-management and bolster their confidence in overcoming the challenges posed by the disease. ③ On a weekly basis, imperative postoperative lymphedema prevention information, encompassing subjects like cutaneous care, rehabilitative exercises, and complication mitigation, is systematically propagated through either the WeChat group or the WeChat public account. Concurrently, personalized adjustments to content are made by referencing entries in the bedside nurse-patient communication book and addressing concerns identified during subsequent follow-up visits. This individualized strategy ensures the provision of pertinent information tailored to the specific needs of each patient. The dissemination of information is executed through captivating illustrations and cartoons, strategically designed to stimulate active patient engagement and compliance. The overarching objective of this approach is to augment patients’ proficiency in postoperative lymphedema prevention knowledge and cultivate adherence to self-management practices. ④ The psychological counseling team underscores the pivotal role of negative emotions in influencing a patient’s prognosis. They advocate for the cultivation and sustenance of an optimistic emotional state as imperative for enhancing treatment outcomes and mitigating complications. In instances where a patient manifests anxiety and depression, stemming from apprehensions regarding the potential impact of lymphoedema on future self-care capabilities, it is recommended to furnish comprehensive information. This should encompass the elucidation of risk factors associated with lymphoedema, standard preventive measures, and the significance of integrating rehabilitation exercises into daily routines as a prophylactic measure against lymphoedema. Conversely, if a patient experiences anxiety and depression unrelated to lymphoedema, proactive measures are advised to encourage the patient to articulate and discuss their concerns openly. Providing targeted and empathetic responses becomes essential in alleviating the negative emotional state of the patient. ⑤ Conduct regular weekly follow-up visits with patients utilizing diverse communication modalities, including telephone, WeChat, and relevant mobile applications. Systematically document identified issues in the follow-up register to facilitate comprehensive tracking and management. During these follow-up interactions, inquire about patients’ behavioral habits, and adherence to prescribed rehabilitation exercises, and assess the functional recovery of the affected limb post-discharge. Promptly institute adjustments to the intervention plan based on the patient’s evolving clinical status. Emphasize the paramount importance of preventative measures against lymphoedema, advocate for protective strategies for the affected limb, and underscore the consistent incorporation of rehabilitation exercises into daily routines. Provide vigilant supervision to ensure patients execute lymphatic reflux exercises in a punctual manner and the correct quantity. Additionally, instruct patients to promptly seek follow-up attention in the event of any discomfort experienced during the training process.

##### 2.2.2.6. Re-observation and reflection.

During this iteration, patients exhibited a heightened and comprehensive understanding of postoperative lymphoedema prevention, leading to an amelioration in their motivation for both learning and self-management. Notably, patients availed themselves of instructional videos disseminated through the WeChat group or WeChat public platform, facilitating the timely and appropriate completion of prescribed rehabilitation exercises, specifically lymphatic reflux exercises. This resulted in a substantial enhancement in patients’ adherence to preventative measures. Actively participating in the process, patients proactively articulated uncertainties, discussed encountered challenges and elucidated the origins of their anxiety and depression. Seeking proactive engagement, patients actively sought support and assistance from healthcare professionals, including medical and nursing staff, the psychological counseling team, as well as their familial network. This concerted effort culminated in heightened patient confidence and motivation to surmount the challenges posed by the disease. These positive outcomes underscore the efficacy of the nursing intervention grounded in the action research method, indicating a more desirable impact, thereby concluding the study.

#### 2.2.3. Observation indicators.

##### 2.2.3.1. Incidence and degree of lymphoedema.

The assessment of lymphedema severity in this study adhered to the measurement protocol outlined in the pertinent literature, specifically as detailed in reference.^[22]^ The evaluation involved measuring the circumference of both the affected and unaffected upper arms at a location 10 cm above the transverse wrist line. To ensure accuracy, measurements were conducted thrice, and the results were subsequently averaged for analysis. The circumference of the affected upper limb is not different from that of the healthy side, indicating no edema; The circumference of the affected upper limb is thicker than the healthy side but within 3 cm, indicating mild edema; The circumference of the affected upper limb is 3 to 6 cm thicker than the healthy side, indicating moderate edema; If the circumference of the affected upper limb is more than 6 cm thicker than the healthy side, and there is a restriction of shoulder joint movement and problematic skin, this indicates severe edema.

##### 2.2.3.2. Ability to perform activities of daily living.

The evaluation of the patient’s ability to perform activities of daily living was conducted using the Barthel Index Scale.^[23]^ This scale comprises 10 items, each scored within the range of 0 to 10, resulting in a total score ranging from 0 to 100. A higher score indicates a better ability of the patient to perform activities of daily living.

##### 2.2.3.3. Anxiety and depression.

The Hospital Anxiety and Depression Scale (HADS) was administered to the patients by the researcher both before and at the conclusion of the study.^[24]^ The scale employed in this study served as a tool for evaluating the psychological well-being of patients. It comprised 2 distinct subscales, namely anxiety and depression, each encompassing 7 items. The total scale encompassed 14 items, graded on a scale ranging from 0 to 3. Notably, elevated scores on this scale were indicative of a more compromised psychological status in the patients under evaluation.^[25]^ The internal consistency coefficients, as measured by Cronbach α, for the total scale, anxiety subscale, and depression subscale were 0.89, 0.76, and 0.83, respectively.^[26]^

#### 2.2.4. Data collection and processing methods.

After the study, the circumference of both affected and healthy limbs was measured in each group using an inelastic dipstick. The resulting difference was utilized to ascertain the presence of edema and determine its severity. The patient’s ability to perform daily activities was evaluated using the Barthel Index Scale. Data analysis was conducted using SPSS 22.0 statistical software, and measurement data conforming to normal distribution were presented as mean ± standard deviation (±standard deviation).^[27]^ The independent samples t-test was employed to compare differences between the 2 groups. Statistical data were presented as counts and percentages (%), and the Chi-square test (*X*^2^) was utilized to assess differences between the groups.^[28]^ A significance level of *P* < .05 was considered indicative of statistical significance.

## 3. Results

### 3.1. Comparing the incidence and severity of postoperative lymphoedema between the 2 groups

The observation group exhibited a lower incidence and severity of postoperative lymphoedema compared to the control group, with the difference being statistically significant (*P* < .05); refer to Table 1 for details.

**Table 1:** Comparison of the incidence and severity of postoperative lymphoedema in the 2 groups.

Group	Number of cases	Number of edema cases	Mild	Moderate	Severe
Observation group	44	9(20.45%)	6(13.95%)	3(6.98%)	0(0.00%)
Control group	44	19(43.18%)	5(11.11%)	9(20.00%)	5(11.11%)
*X*^2^-value		5.238	–	–	–
*P*-value		0.022	–	–	–

### 3.2. Comparison of daily living ability between the 2 groups of patients before and after the study

At the commencement of the study, there was no statistically significant difference in the Barthel Index Scale scores between the 2 patient groups (*P* > .05). By the study’s conclusion, both groups demonstrated improvement in Barthel Index Scale scores. Notably, the observation group displayed higher scores compared to the control group, with the difference being statistically significant (*P* < .05); refer to Table 2 for details.

**Table 2:** Comparison of daily living skills between the 2 groups of patients before and after the study ($\bar{x}\pm s$).

Group	Number of cases	Pre-study scores	Post-study score
Observation group	44	52.63 ± 5.28	82.36 ± 4.55*
Control group	44	51.51 ± 6.02	77.18 ± 2.92*
*t*-value	–	0.924	6.431
*P*-value	–	0.358	0.000

* *P* < .05 compared to pre-study.

### 3.3. Comparison of anxiety and depression scale scores between the 2 groups of patients before and after the study

At the initiation of the study, there was no statistically significant difference in the HADS anxiety and depression subscale scores between the 2 patient groups (*P* > .05). By the study’s conclusion, both groups exhibited a decrease in anxiety and depression scores. Remarkably, the observation group demonstrated lower anxiety and depression scores compared to the control group, with the difference being statistically significant (*P* < .05); refer to Table 3 for details.

**Table 3:** Comparison of HADS scores between the 2 groups of patients before and after the study ($\bar{x}\pm s$).

Group	Number of cases	Anxiety score		Depression score	
		Pre-study	Post-study	Pre-study	Post-study
Observation group	44	18.70 ± 1.40	11.36 ± 1.17*	17.40 ± 1.58	12.62 ± 0.85*
Control group	44	18.73 ± 1.45	16.82 ± 1.45	17.42 ± 1.57	16.28 ± 1.50
*t*-value	–	−0.136	−20.180	−0.072	−18.159
*P*-value	–	0.898	0.000	0.942	0.000

* *P* < .05 compared to pre-study.

## 4. Discussion

### 4.1. Action research-based care reduces the incidence of postoperative lymphoedema in breast cancer

Lymphoedema is the most common complication after breast cancer surgery. Once it occurs, it is likely to progress from mild to severe edema, often requiring lifelong management, making early prevention of postoperative lymphoedema essential in the clinical workup.^[29]^ Guidelines and consensus recommendations for postoperative lymphoedema after breast cancer recommend that lymphoedema focuses on lifelong self-care practices to maintain arm health and minimize associated complications.^[30]^ There are various models of care for the prevention of postoperative lymphoedema after breast cancer in clinical practice. Still, most of them are passively accepted by patients, with a single approach and lack of systematization, which cannot meet the current clinical needs of postoperative breast cancer patients, resulting in a less than satisfactory overall care effect.^[12]^ This study employed the measurement of upper arm circumference on both the affected and healthy sides, utilizing the disparity to assess the occurrence and severity of edema. However, the intervention process involved surgical procedures, chemotherapy, and radiotherapy, precluding a direct comparison of effects before and after intervention under identical physical conditions. Consequently, potential biases may have influenced the outcomes. The incidence of lymphoedema in the observation group demonstrated a gradual reduction post-intervention, with all levels of lymphoedema exhibiting a statistically significant decrease compared to the control group (*P* < .05). This implies that the action research-guided intervention program, augmenting traditional nursing measures, yielded favorable results. The findings suggest a notable reduction not only in the incidence of postoperative lymphoedema in breast cancer patients but also in the severity of edema. This positive outcome can be attributed to the innovative nature of action research-based nursing, characterized by its problem-centric approach involving collaborative engagement of practitioners and researchers. Employing a 4-step spiral cyclic process of planning, action, observation, and reflection, this method effectively addresses practical issues during clinical practice, rendering the nursing program more scientific and standardized. In this investigation, an in-depth exploration of the challenges faced by breast cancer patients in averting lymphoedema post-surgery was undertaken through structured interviews. Subsequently, based on the identified difficulties, precise intervention strategies and implementation programs were developed. Scientific guidance was consistently provided throughout the entire continuum of care, complemented by a dynamic assessment approach incorporating patient participation. Timely resolution of nursing-related issues ensured active patient engagement in lymphoedema prevention measures. Within the observation group, a multimedia approach was adopted to impart lymphatic reflux exercise techniques to patients, utilizing video presentations and illustrative materials. Concurrently, during the initial phase of the intervention cycle, patients received multifaceted education to deepen their understanding of lymphoedema prevention. The subsequent cycle involved a comprehensive review of issues identified in the previous phase, with subsequent refinements made. Post-discharge, personalized guidance, and the establishment of a WeChat group facilitated the dissemination of knowledge and operational instructions related to lymphoedema prevention through the WeChat application. This prompted patients to adhere to prescribed routines, submit regular updates, and seek real-time online assistance for encountered challenges. The integration of an online platform facilitated not only the resolution of uncertainties through timely question-and-answer sessions but also the establishment of a collaborative learning environment among patients. This interactive approach significantly bolstered patient compliance with self-preventive measures against lymphoedema, thereby contributing to a notable reduction in the incidence of this condition.

### 4.2. Action research-based care can improve self-care in post-breast cancer patients

The findings derived from this investigation revealed that, upon conclusion of the study, patients within the observation group exhibited superior self-care scores in comparison to those in the control group. This discrepancy suggests that care interventions grounded in the action research method yielded notable enhancements in self-care practices subsequent to breast cancer surgery.^[31]^ The underlying rationale for this phenomenon may be attributed to the application of the action research methodology as an intervention. Following the implementation of this method, patients exhibited an enhanced comprehension of lymphoedema-related knowledge, thereby diminishing the risk of lymphoedema occurrence through proactive preventive measures. Consequently, there was a discernible cessation in the progression of lymphoedema, accompanied by a gradual amelioration of associated symptoms. This ameliorative trajectory, in turn, alleviated the physiological discomfort experienced by patients and concurrently fortified their confidence in the recovery process. The action research method is practice-oriented and provides health guidance to breast cancer patients in the course of action. It observes and reflects on patients’ behavior, enabling them to participate more actively in health education. Members of the research team guided patients on risk factors, prevention methods, and rehabilitation exercises through various forms such as WeChat groups and WeChat public numbers. On the one hand, the efficiency and effectiveness of health education were improved. On the other hand, breast cancer patients’ compliance and motivation to care measures increased. The re-action in the cycle includes regular follow-up visits, which can help patients develop scientific and standardized out-of-hospital rehabilitation methods according to their behavioral habits and actual conditions, and supervise patients to perform lymphatic reflux exercises on time and in the right amount, which improves patients’ awareness of self-management and enables them to cope with changes in their lifestyle due to the disease in a positive way, thus enhancing their ability to take care of themselves.

### 4.3. Action research-based care alleviates anxiety and depression in postoperative breast cancer patients

Anxiety and depression are common in oncology patients, and postoperative breast cancer patients experience significantly higher levels of anxiety and depression due to surgical stress, menopause, mastectomy, and chemotherapy side effects.^[11,13]^ Postoperative breast cancer patients suffer from anxiety, depression, low self-esteem, and other psychological problems due to the absence of the breast, which destroys the integrity of the body and affects the unique gender charm of women.^[32]^ At the end of this study, the anxiety and depression scores of patients in the observation group were lower than those of the control group, suggesting that action research method-based care could alleviate the anxiety and depression of postoperative breast cancer patients. The underlying cause for this phenomenon can be attributed to the utilization of the action research method, which encompasses a two-round cycle involving multimedia and graphic education, psychological guidance, and family support. This comprehensive approach not only facilitates the acquisition of enhanced knowledge pertaining to lymphoedema prevention among breast cancer patients but also fosters familial bonds and mitigates negative emotions. Consequently, this multifaceted intervention contributes to heightened patient confidence and improved psychological resilience. Furthermore, within the paradigm of action research method-based care, a specialized psychological counseling team was instituted during the re-action phase. The designated counselor adeptly discerned patients’ adverse emotional states in a timely fashion throughout the intervention trajectory, concurrently imparting efficacious psychological interventions to assist patients in coping with and alleviating these emotions. Notably, the intervention demonstrated effectiveness in diminishing the severity of edema in the affected limbs and ameliorating associated symptoms. This outcome directly engendered a sense of hope among patients, augmenting their vitality. The dissemination of successful cases within the institutional setting, as well as the reciprocal encouragement among patients in the extramural WeChat group, collectively contributed to fostering confidence, mitigating anxiety and depression, and facilitating effective emotional management among BCRL patients. This collective effort guided patients toward maintaining a positive and optimistic disposition, thereby reducing their psychological burden and alleviating anxiety and depression.

## 5. Conclusion

In conclusion, care based on action research methodology has demonstrated efficacy in reducing the incidence of postoperative lymphoedema, enhancing the self-care ability of postoperative breast cancer patients, and alleviating anxiety and depression. These positive outcomes warrant promotion and application in clinical care. Nevertheless, this study has certain limitations and shortcomings. Firstly, being a single-center study with a limited number of cases, it falls short of achieving a large sample selection across multiple geographical areas. This limitation may introduce bias into the study results. Future validation of these findings requires a multi-center large sample study to enhance the generalizability and robustness of the results.

## Author contributions

**Conceptualization:** Weijuan Yang, Lili Wang.

**Data curation:** Weijuan Yang, Li Yang, Shuangwei Mao, Dandan Liu, Lili Wang.

**Formal analysis:** Weijuan Yang, Lili Wang.

**Funding acquisition:** Weijuan Yang, Lili Wang.

**Investigation:** Weijuan Yang, Li Yang, Shuangwei Mao, Dandan Liu.

**Methodology:** Weijuan Yang.

**Project administration:** Weijuan Yang.

**Resources:** Weijuan Yang.

**Software:** Weijuan Yang.

**Supervision:** Lili Wang.

**Validation:** Lili Wang.

**Visualization:** Weijuan Yang, Lili Wang.

**Writing – original draft:** Weijuan Yang.

**Writing – review & editing:** Weijuan Yang, Lili Wang.
